# An exploratory study of treated-bed nets in Timor-Leste: patterns of intended and alternative usage

**DOI:** 10.1186/1475-2875-10-199

**Published:** 2011-07-21

**Authors:** Andrew A Lover, Brett A Sutton, Angelina J Asy, Annelies Wilder-Smith

**Affiliations:** 1 Department of Epidemiology and Public Health Yong Loo Lin School Of Medicine National University of Singapore MD3, 16 Medical Drive, 117597, Singapore; 2 Timor-Leste Asisténcia Integradu Saúde (TAIS), (Timor-Leste Integrated Health Assistance), Bario Formosa Dili, Timor-Leste; 3 Institute of Public Health, University of Heidelberg Im Neuenheimer Feld 365 69120 Heidelberg, Germany

## Abstract

**Background:**

The Timor-Leste Ministry of Health has recently finalized the National Malaria Control Strategy for 2010-2020. A key component of this roadmap is to provide universal national coverage with long-lasting insecticide-treated nets (LLINs) in support of achieving the primary goal of reducing both morbidity and mortality from malaria by 30% in the first three years, followed by a further reduction of 20% by end of the programme cycle in 2020 [1]. The strategic plan calls for this target to be supported by a comprehensive information, education and communication (IEC) programme; however, there is limited prior research into household and personal usage patterns to assist in the creation of targeted, effective, and socio-culturally specific behaviour change materials.

**Methods:**

Nine separate focus group discussions (FGDs) were carried out in Dili, Manatuto, and Covalima districts, Democratic Republic of Timor-Leste, in July 2010.

These focus groups primarily explored themes of perceived malaria risk, causes of malaria, net usage patterns within families, barriers to correct and consistent usage, and the daily experience of users (both male and female) in households with at least one net. Comprehensive qualitative analysis utilized open source analysis software.

**Results:**

The primary determinants of net usage were a widespread perception that nets could or should *only *be used by pregnant women and young children, and the availability of sufficient sleeping space under a limited number of nets within households. Both nuisance biting and disease prevention were commonly cited as primary motivations for usage, while seasonality was not a significant factor. Long-term net durability and ease of hanging were seen as key attributes in net design preference. Very frequent washing cycles were common, potentially degrading net effectiveness. Finally, extensive re-purposing of nets (fishing, protecting crops) was both reported and observed, and may significantly decrease availability of nighttime sleeping space for all family members if distributed nets do not remain within the household.

**Conclusions:**

Emphasizing that net usage is acceptable and important for all family members regardless of age or gender, and addressing the complex behavioural economics of alternative net usages could have significant impacts on malaria control efforts in Timor-Leste, as the country's programmes make progress towards universal net coverage.

## Background

Malaria has been a major source of morbidity and mortality for all of recorded history. While it has been well controlled in many developed nations, it remains the major infectious disease in many regions of the world. For 2008, the WHO estimates 243 million (95% CI, 190 million to 311 million) global infections, causing 863,000 deaths (95% CI, 708,000 to 1,003,000); Southeast Asia contributes 10% of these infections, and 5% of the global mortality [2].

In the past decade several large initiatives have made malaria control one of their primary goals, particularity the Global Fund, and the Roll-Back Malaria Programme. There have also been renewed calls to make malaria eradication a global priority [3]. Together, these initiatives have greatly increased both awareness and the number of intervention programmes, which has allowed many countries to progress towards achieving Millennium Development Goal number 6. The benchmark for this goal is to reduce by 2015 malaria transmission rates by two-thirds from their 1990 levels [4].

### Malaria in Timor-Leste

This study focuses on malaria in Timor-Leste. Timor-Leste, Asia's newest nation, has in the past decade made major advances in rebuilding its shattered health systems after a protracted independence process.

However, malaria is still one of the most significant health issues in the country: 100% of the country's residents are at year-round risk [5]. The most recent survey data suggests that the reported malaria prevalence is increasing throughout the country: from 2000 to 2007 reported cases rose from 113 to 207 per 1,000 [6]. The primary driver of this change was greatly improved diagnostic capabilities (both microscopy and rapid tests) throughout the country. The best estimate for morbidity associated with infections is > 97,000 probable cases per annum (2008); the most heavily affected regions have an annual parasitaemia index (API) of > 250 (confirmed cases per 1,000 population per year) [7]. However, only about 20% of suspected infections are confirmed via microscopy or rapid tests, so this number represents a potential underreporting of the true prevalence [5].

Of the confirmed infections, about 70% are *Plasmodium falciparum*, and 30% are *Plasmodium vivax*; with mixed infections also common in many areas, and variable proportions across the districts [1]. A recent published cross-sectional parasitaemia survey suggests that there is a wide range in prevalence by geography, and the authors report that malaria is most strongly associated with patient age, type of housing construction, ITN usage and "exposure to mosquitoes" [8,9].

There are no reliable national estimates for mortality: in 2009, the Timor-Leste Ministry of Health reported a total of 53 deaths from malaria [7]. All of these deaths occurred specifically at the National Hospital in Dili. There are no vital registry systems in Timor-Leste, so the vast majority of deaths occur outside any reporting structures.

The Millennium Development Goal target prevalence rate for 2015 is 45 per 1,000 population; it is unlikely, however, that Timor-Leste will be able to make rapid enough progress to meet this goal [4].

### Insecticide-treated bed nets as a key intervention

A wide range of tools have been developed to impact the epidemiology of malaria, including household insecticide treatments; strengthening community health systems for prompt and correct treatment of infections; the use of fish to control mosquito larvae; and presumptive treatment of pregnant women to decrease parasite transmission. The most cost-effective community strategy has been the use of an insecticide-treated net or ITN [10,11]. The *Anopheles *sp. mosquito vectors that transmit malaria feed only at night, so nighttime coverage under an ITN is an uniquely effective intervention. Secondly, the insecticidal effect kills mosquitoes that land on the nets, removing them from any potential transmission. This mass action effect is the single factor that makes treated-bed nets significantly more effective than untreated models [12,13].

The health impact of treated-bed net usage has been validated in a wide range of transmission settings [14,15]. Correct and consistent use of nets can decrease transmission in a community setting by 90%, and can lower all-cause under-5 mortality by 44% [16]. Net usage also has significant impacts on pregnancy outcomes for both the mother and the neonate by greatly reducing maternal anaemia and placental insufficiency, leading to lower risk of complications and fewer premature or underweight births [11,16].

One of the factors that contribute to this success is the relatively low cost, ease of use, and simplicity of distribution; approximately 250 million nets have been distributed worldwide in recent years [2].

However, even in countries with very high levels of household ownership, nighttime net usage is often low. Many studies have explored the dynamics of this disparity, especially in sub-Saharan Africa. These studies have identified a wide range of factors in different environments; the most common of these include users reporting being too hot under nets, difficulties hanging nets in traditional houses, and not having enough space under nets for all household members [17,18].

### Timor-Leste: current ITN usage

There are currently three types of nets in widespread use in Timor-Leste: simple untreated nets, re-treatable insecticidal nets (ITNs), and long-lasting insecticide-treated nets (LLINs). The most basic untreated nets are commonly purchased at community markets, and have limited lifespan and epidemiologic impact. Re-treatable nets were the standard distribution for many years; these nets required re-treatment at six-month intervals with an insecticide solution. Compliance with re-treatment schedules was generally very poor, so distribution of these was phased out in 2006 throughout Timor-Leste. However, many of these nets are still in daily use and are counted in the usage totals, even though re-treatment packs are unavailable anywhere in the country.

All recent, and current campaigns distribute LLINs, which have the insecticide impregnated into the fabric during manufacture. These products retain protective effects for the entire 3-5 year lifetime of the net. Three brands have been distributed in Timor-Leste: Olyset (Sumitomo, Japan), Permanet (Vestergaard, Denmark) and Dawa-Plus (TanaNet, Thailand), all in a variety of colours and configurations. The Olyset and Permanet have a projected lifespan of five years; Dawa-Plus are designed for three. Due to funding considerations, the current distribution programmes in Timor-Leste all focus on the Dawa-Plus, which will need to be replaced sooner than other brands.

Nets are freely distributed through antenatal clinics, and the National Malaria Control Programme has also partnered with several NGOs to deliver nets in other campaigns that specifically target pregnant women and children under-five. Hundreds of thousands of nets have been freely distributed in Timor. From 2007 to 2009, 175,000 ITNs and LLINs were distributed to pregnant women and children under-five, providing coverage for just over 30% of these high-risk population [5]. The 2010 Demographic and Health Survey (DHS) reported approximately 80% usage the night before; this current coverage and prior-night usage data is summarized in Table 1[19].

**Table 1:** Data from the 2010 Demographic and Health Survey (DHS)

Surveyed Characteristic	Percentage
Net Ownership (at least one ITN)	
Rural	37.7
Urban	51.0
Wealth, lowest quintile	23.6
Wealth, highest quintile	55.2

ITN Usage (previous night)	
Children under-5 (all households)	41.0
Children under-5 (ITN owners)	83.0
Pregnant women (all households)	40.7
Pregnant women (ITN owners)	84.4

While these numbers suggest very high levels of use the previous night among households that own nets, the most important metric, "correct and consistent usage," was not captured in this survey. Data from other studies in Southeast Asia suggests that real-world usage is often significantly different from self-reports during surveys. For example, in Vietnam, survey data reported 92% use the night before, with 86% reporting all family members had slept under an ITN or LLIN. However, the investigators also performed unannounced nighttime visits to the same villages and households, and observed that there were no nets in use in 20% of houses, and many nets that were being used did not reach the floor or were being used as blankets, and so provided little or no protection [20].

### Timor-Leste: barriers to usage (gap analysis)

A large number of studies addressing barriers to ITN usage in sub-Saharan Africa have been published, but comparatively few in Asia. A single study was performed in Timor-Leste in 2006, but was specific for net usage during an epidemic within a camp run by the UN for internally displaced persons (IDPs) outside of the capital city, Dili [21]. This multi-mode study included three focus groups: one for IDP camp managers, one for health staff, and a single discussion for net users. The main conclusions were uncertainty about net usage and effectiveness, and whether or not distributed nets were actually utilized. However there have been no detailed studies published on usage patterns or user acceptability in Timor-Leste.

### Study objectives

The primary goal was to explore the nightly usage patterns, the levels of consistent and correct usage of treated-bed nets, and to explore any barriers to usage. Specifically, we aimed to investigate the patterns of behaviour, decision-making processes within households, general levels of risk awareness, and to explore any net design issues that might contribute to the observed usage patterns.

## Methods

### Study areas and target demographic

This study was conducted in July 2010, which is generally the driest part of the year in Timor-Leste; however, the island had extensive rains and flooding during this season in 2010. The calculation of sample size is not a significant issue in qualitative research. The general guideline is that focus groups should be undertaken until the responses are "saturated" [22]. This occurs when the same responses are repeated over and over in various settings, suggesting that the concepts have been fully explored. In planning this study, the need to reach saturation was balanced with a motivation to explore any geographic variations in ITN usage patterns within Timor-Leste. There was also a limitation to work in areas which were accessible, open to the possibility of focus groups, and where local contacts existed.

**Figure 1:**
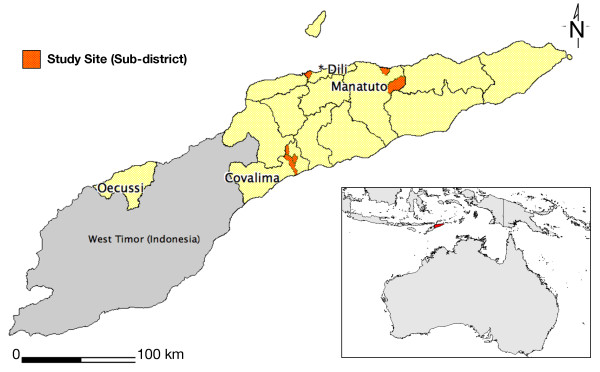
**Location of study areas**.

After considering these factors and consulting experts at the National Malaria Control Programme, three different districts were chosen as study sites for a total of nine separate focus groups: Dili, Manatuto, and Covalima. These regions were selected due to high malaria transmission patterns, as well as having a wide range of climates and ethnic groups. The study areas are shown in Figure 1.

Due to the strict gender roles within Timor-Leste and the potentially sensitive nature of household sleeping patterns, focus groups for men and women were run independently of one another. This allowed ideas to be shared with minimal social pressure on the respondents.

**Figure 2:**
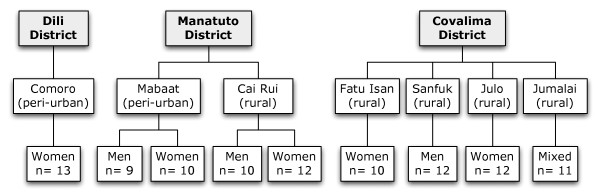
**Overview of focus groups**.

In Dili, there was a single discussion for women; there were four discussions in Manatuto (two for men and two for women, paired in two separate villages); and four focus groups in Covalima (one for men, two for women and one mixed, in four separate villages). (Figure 2).

These study sites fell along a continuum of development and access to services; however, malaria is prevalent throughout the country even in urban areas. The site in Dili district (Comoro *aldeia*) is a village on a peri-urban fringe, but retains good access to health services, including regular health care worker visits for vaccination campaigns. The sites in Manatuto district (*Suco *Mabaat, *Suco *Cai Rui) included both an area close to the town of Manatuto, and a rice farming area with significantly fewer services. Covalima district is one of the least developed areas of the country, but the sites (*Aldeia *Fatu Isin, *Aldeia *Sanfuk, *Suco *Julo, *Suco *Tasihilin) all had some limited access to health services. Additionally, the region has benefited from a significant NGO presence. (*Suco *is a neighbourhood in urban areas or a rural village; *aldeia *is a rural hamlet.)

The target demographic consisted of caregivers and heads-of-households between the ages of 18 and 49 from households that owned at least one net, of any type. The study population was a convenience sample, recruited through a network of local public health staff and local village leaders. There was no compensation for their participation, but snack foods and bottled water were supplied at all discussion groups.

### Development and use of instruments

A short survey in Tetun, the primary local language, was developed to capture basic demographic details of all study participants. Participants, who were able to, filled in the forms on their own; primary centre health staff or the Timorese study facilitators assisted those with lower literacy levels. All completed forms were checked on-site and corrected when typographic or comprehension errors were noted.

The development of the focus group (FG) guide was an iterative process, using local experts and the report from a Red Cross project addressing malaria behaviours in rural Cambodia [23]. In consultation with the Timor-Leste Ministry of Health, John Snow Inc., and the Manoff Group, these general outlines were then adapted to fit both the specific questions to be probed in Timor, and to ensure social and cultural relevance. Following this, preliminary field-testing and revision occurred.

The FG discussion guide, and a short set of guidelines for the facilitators were then translated into Tetun, and the translated version was checked for linguistic and concept consistency by three bilingual collaborators. A set of test runs was performed with local staff, and then revised. This preliminary FG guide was used to test the real-world performance of the instrument, which led to minor revisions.

### Informal bed net surveys and interviews

To triangulate focus group data, a set of informal net surveys was carried out at three discussion sites with sufficient housing in proximity to the FGD, using any volunteers (both men and women) who would consent to a home visit by the study staff. Informal, *ad hoc *interviews were also conducted with local leaders, shopkeepers where nets were sold, and governmental/NGO staff involved in net distribution programmes.

### Ethics

Ethics review for this study was submitted to the Timor-Leste Cabinet of Health Research and Development at the Institute of Health Sciences (ICS); full approval was granted on 1 June 2010. Individual informed consent was obtained from each participant following verbal explanation of all study aims, procedures, and confidentiality of data.

### Field study staff, procedures and data management

All FG facilitators were local Timorese public health staff with prior focus group experience, and included one man and three women. They were orientated to the guidebook and detailed aims of the study. Note-takers for the focus groups were also local health staff who were familiar with all malaria and health-related terms encountered. In addition to note taking, all focus groups were recorded simultaneously on two digital recorders. The facilitators directly addressed any potentially serious gaps in health knowledge and practice at the conclusion of each focus group, and also provided linkages with local primary health care services.

### Data analysis

The digital recordings were transcribed and translated from Tetun into English by local health staff. The transcripts and notes from the focus groups were then analysed using TAMS (Text Analysis Markup System) Analyzer software (version 3.0, open source) [24]. Using a paradigm of "grounded coding" 28 different categories were created after exhaustive re-reading of the text [25]. Clarification of all unclear sections was obtained from the translators. These categories were then utilized to code all of the text, and output files for each category were created. These grouped entries were then re-examined, and the dominant responses were summarized and combined with illustrative quotes. After coding of all the text, the transcript was then re-read with the issues that emerged in mind, and re-coding performed as necessary.

## Results and discussion

The size of the focus group discussions ranged from eight to thirteen participants, with an age range of 18 to 79 for men, and 18 to 78 for women, with few individuals over 60. The initial target age range of 18 to 49 was chosen to capture the demographic most likely have young children in the household; however, the convenience sample selected by village leaders was not always within this range. A range of basic socio-economic and demographic indicators was collected prior to the start of all of the discussion groups (Table 2). The study population was broadly similar to national level demographics, as reported in the 2010 DHS survey [19].

**Table 2:** Study Population Demographics

Characteristic	Females		Males		Total	
	**n**	**% of total**	**n**	**% of total**	**n**	**% of total**

Age (yrs)						
18-24	10	18.9	11	23.9	21	21.2
25-29	10	18.9	5	10.9	15	15.2
30-39	16	30.2	13	28.3	29	29.3
40-49	12	22.6	6	13.0	18	18.2
40-59	1	1.9	4	8.7	5	5.1
60+	4	7.5	7	15.2	11	11.1

Primary occupation						
Homemaker or childcare	48	90.6	11	23.9	59	59.6
Farmer or fisherman	2	3.8	23	50.0	25	25.3
Manual labour	0	0.0	8	17.4	8	8.1
Shopkeeper, etc.	1	1.9	1	2.2	2	2.0
Student	0	0.0	0	0.0	0	0.0
Other	2	3.8	3	6.5	5	5.1

Education						
No schooling	13	24.5	15	32.6	28	28.3
Primary	16	30.2	10	21.7	26	26.3
Pre-secondary	14	26.4	10	21.7	24	24.2
Secondary	8	15.1	10	21.7	18	18.2
University	2	3.8	1	2.2	3	3.0

Number of children						
0	3	5.7	6	13.0	9	9.1
1 to 2	12	22.6	13	28.3	25	25.3
3 to 4	20	37.7	14	30.4	34	34.3
5 to 6	9	17.0	11	23.9	20	20.2
7 to 8+	9	17.0	2	4.3	11	11.1

Age of Youngest Child						
n/a	3	5.7	6	13.0	9	9.1
Under 6 months	4	7.5	4	8.7	8	8.1
6 months to 2 years	13	24.5	11	23.9	24	24.2
3 to 4 years	15	28.3	7	15.2	22	22.2
5 to 6 years	4	7.5	7	15.2	11	11.1
7 to 8 years	4	7.5	0	0.0	4	4.0
9 to 10+ years	11	20.8	11	23.9	22	22.2

**Total**	n = 53		n = 46		N = 99	

### Selected participant responses

A range of opinions, ideas and attitudes emerged from the nine focus groups; representative quotations illustrate common themes in focus groups for both men and women (Tables 3 and 4).

**Table 3:** Selected Illustrative Comments I

Topic	Quote	District
Causes of malaria	"Littering and rubbish everywhere." (Woman)	Dili
	"According to me the flies also could produce malaria if we did not keep our food safely on the table while ready to eat." (Woman)	Manatuto
	"Not neatly folding the clothes." (Woman)	Manatuto
	"Malaria is from mosquitoes, rubbish, and flies." (Man)	Manatuto
	"Many germs in the water tanks" (Woman)	Covalima

ITN Effectiveness	"Yes, they protect us from malaria." (Men and women)	All sites
	"No! They do not protect us. Although we use the nets, we are still exposed to malaria!" (Woman)	Dili
	"We have used mosquito nets for a long time, but we are always out of house in the garden." (Woman)	Covalima

ITN Usage, Likes and Dislikes	"For us it is hot, but we need to use it anyway." (Woman)	Dili
	"My father, he does not like it because the net always challenges him when waking up on the morning." (Woman)	Manatuto
	"I do not feel comfortable because the net is too small." (Man)	Manatuto
	"No problem- for us living in the mountains, we feel comfortable using mosquito nets." (Man)	Manatuto
	"Our husbands like it because they are also scared of mosquitoes." (Woman)	Covalima
	"Oh, no one does not like to sleep in a mosquito net! Because if you do not use mosquito nets when sleeping, the mosquito's voice will make us not sleep well and does not make it safe to sleep." (Woman)	Covalima
	"Yes, it is very easy use- no problem." (Man)	Covalima
	"We like it because the mosquito can not bite us." (Man)	Covalima

**Table 4:** Selected Illustrative Comments II

Topic	Quote	District
ITN decision- making within households	Query: "Who decides which household members sleep under the nets you own?"	
	"The chief of the family, or it could be other members of the family." (Woman)	Dili
	"Mothers are the ones that make decisions for the kids." (Women)	Dili
	"Both the husband and the wife decide these things." (Woman)	Dili
	"The doctors decide for us." (Man)	Manatuto
	"...the man makes the decisions." (Man)	Manatuto
	"The man is the chief of the family." (Women)	Covalima
	"...because the man is the chief of the family" (Men)	All sites

ITN Usage, beliefs and patterns	"Nets are only for young children." (Men and women)	All sites
	"The mosquito nets separated couples to sleep in different rooms." (Woman)	Manatuto
	"The mosquito net is only for pregnant mothers and it is not for fathers." (Women)	Manatuto
	"As far as I am concerned two family members only can use mosquito nets- they are the mother and the child." (Man)	Manatuto
	"We are not to use nets, but we use them when we feel cool. The doctor also mentioned that it's not for the man." (Man)	Manatuto
	"Depending on the bed net, it could be two, three, four, five up to six people sleeping under it." (Women)	Covalima
	"Fathers cannot sleep inside the mosquito net if the mother is pregnant or has a small baby." (Man)	Covalima

### Primary themes

The set of nine focus groups provided a rich tapestry of experiences within Timor-Leste. The study not only covered issues relating to treated net usage, but also social dynamics, family patterns, economic pressures, and general attitudes towards health.

In most villages, malaria was *the *largest health concern, among both men and women, in rural and peri-urban areas, and most respondents were aware of the benefits of sleeping under a net. However, a wide range of alternative uses for the nets was also reported, emphasizing the serious economic calculations families make. Most participants reported very positive experiences with sleeping under nets, and many of these responses mentioned both the reduction of nuisance biting and disease prevention as main motivators.

The most striking result that emerged was uncertainty about which family members could or could not sleep under the nets. One result of this confusion in Timor-Leste was the separation of couples at night, with potentially large implications for long-term user acceptability. The other result is the exclusion of many men and older children from the protection provided by nets already in households.

### Effectiveness of nets

While most users had very positive experiences with nets, a smaller number expressed frustration that malaria was still a problem even with regular net use. One likely contributor to this is the fact that essentially all febrile illness is assumed to be malaria, leading to pervasive confusion about other infectious agents. The common Tetun expression for both malaria and for fever is *isin manas*, (literally 'hot body'), which is an indication of how fever and malaria are perceived as synonymous. Messages that highlight other illnesses that can also cause fever, and the need to seek prompt treatment will remain essential.

Moreover, health campaign posters from the Ministry of Health support the use of treated nets to protect against dengue; this was echoed in several focus groups. These messages have the potential of reducing usage, if it is believed that nets do not protect against malaria or fevers. This highlights the importance of confirmed diagnoses, another key goal of the National Malaria Strategy [1].

### Family dynamics and gender roles

One of the key questions that this study attempted to probe was the dynamics within families about how decisions are made if sleeping space under nets is limited. However, it appears that this topic was too sensitive to get detailed and direct answers. Most of the men answered that they, as "chief of the family" made the decisions; women largely echoed this. A much smaller number suggested that decisions were co-operative, or that the mothers/grandmothers made decisions. The very strict gender roles in Timorese society and the private nature of marriages/relationships, combined with the study facilitators' outsider status, made it very challenging to fully explore these issues.

Overall, the discussions with men were much shorter than with the women, with less rich data. The single exception to this trend was one focus group in Covalima, where the men talked for 90 minutes, and had many detailed responses. The fact that malaria is a constant, and serious problem in this region may explain this differing behaviour. This district also has highly motivated health staff, and has benefited from significant NGO inputs from many organizations, which presumably has impacted the knowledge, attitude and practice levels.

### Usage beliefs

One surprising theme emerged at most of the focus groups- namely, that the nets were *only *for use by pregnant women and young children. Fathers in Covalima and Manatuto said they could not sleep under the nets, and that doing so would be harmful to the baby. Several groups were then asked for clarification, and reported that they had been told by doctors or health providers that the nets were only for these two groups, stating emphatically "... that is based on the information that we got from the Ministry of Health - that the mosquito net is only prioritized to pregnant mother and children under five years old." (Female, Covalima). Several male respondents voiced frustration at sleeping apart, which has a potential of limiting nightly usage. It clearly also creates ongoing risk of nighttime mosquito biting and therefore malaria to men and older children, even where nets are sufficient in number. Where there are insufficient nets, this issue is difficult to manage until Timor-Leste approaches universal net coverage. However some households have sufficient nets for all or most family members and yet still exclude this demographic based on their understanding of the current messages regarding ITN use. Examination of a range of policy statements and implementation guides mirrors this confusion:

"The WHO Global Malaria Programme, therefore, recommends full coverage of all people at risk of malaria in areas targeted for malaria prevention with LLINs. The way in which full coverage should be achieved may vary with particular epidemiological and operational situations. Where young children and pregnant women are the most vulnerable groups, their protection is the immediate priority while progress is made towards achieving full coverage. In areas of low transmission, where all age-groups are vulnerable, national programmes should establish priorities on the basis of the geographical distribution of the malaria burden" [26].

While the programmes in Timor-Leste are fully aligned with these WHO guidelines, the subtlety of conveying messages that prioritize under-fives and pregnant women while making other groups a secondary priority might be simply impractical.

These policy guidelines may be quite difficult to apply to rapidly changing communities; moreover in Timor-Leste there is limited detailed information about the local epidemiology of malaria. However, to achieve maximum impact from limited resources, further research is needed to provide the clearest possible guidance to design optimal behaviour change communication strategies.

### Net preferences

One stated aim of the research was to elucidate preferences in brands, colour or feel of the mesh fabric, or design characteristics that could help to make usage simpler. However, this was difficult, as different brands of nets have been distributed in the same colour scheme (i.e., "the blue kind" could refer to either Olyset or Permanet, depending on the distribution round). In general, the softer feel of Dawa-Plus was perceived as not sturdy enough for extended use, and the stronger, stiffer mesh of the other two brands was seen as preferable, but detailed preferences were not possible with men or women based on their previous experiences.

**Figure 3:**
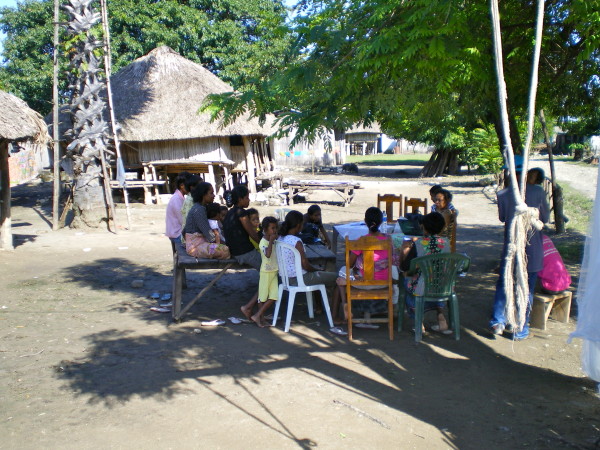
**Women discussing net preferences, Covalima District**.

Several women also noted that the lack of entry doors or flaps in the nets made it difficult for children to get in and out by themselves, and reported that much of the damage to nets was from children. Several groups also commented that they preferred the conical shaped commercial untreated nets, (having a single hang point) to the generally distributed square ones, which require multiple attachment points. (Figure 3).

### Likes and dislikes of net usage

The vast majority of users had a very positive impression about sleeping under the nets. Generally, the avoidance of biting (nuisance effects) was given as a motivator, while explicit disease avoidance was slightly less common. Groups in the cooler mountainous areas reported liking the nets for keeping them warmer, and most people felt that they slept better under the nets.

The few dislikes mentioned were that the nets were too hot, too small, and difficult to enter and exit. One issue was that the net forced couples to sleep in different rooms; this would likely have a serious impact on usage patterns.

### Washing and repairing nets

Many women reported washing their nets every few months or twice a year; however, a significant number reported washing their nets every few weeks, which could severely degrade the performance [27]. Dust was mentioned as one primary reason that children don't like to sleep under the nets; washing frequently could therefore be an effort to increase compliance within the household. Direct observation of the nets currently in use in a convenience sampling of households showed a wide range of conditions, from brand new examples to over 7 year-old treatable nets still in daily use. Timorese households observed in this study generally had dedicated sleeping spaces which included semi-permanent wooden supports for bed nets. As such, these nets did not need to be re-hung everyday, which likely extends the lifespan of nets greatly.

Most women in all the groups said they would re-purpose or even burn older nets with holes, and repair was not a common practice. Many were aware of drying the nets in the shade, but several also mentioned, "retreating with medicine" when these kits are not, and have not, been available in Timor-Leste for several years. This supports at least some respondents trying to give us the "correct" answers for some topics.

### Behavioural economics and alternative net uses

A range of respondents reported knowing of people (often from "neighbouring villages") who used, or had used, their nets for a wide range of other purposes.

**Figure 4:**
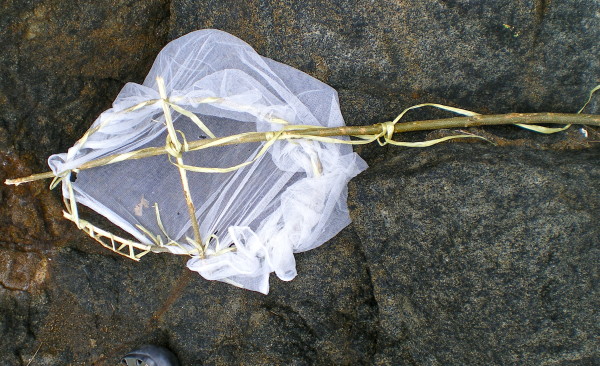
Hand fishing net fashioned from an ITN, Dili District.

**Figure 5:**
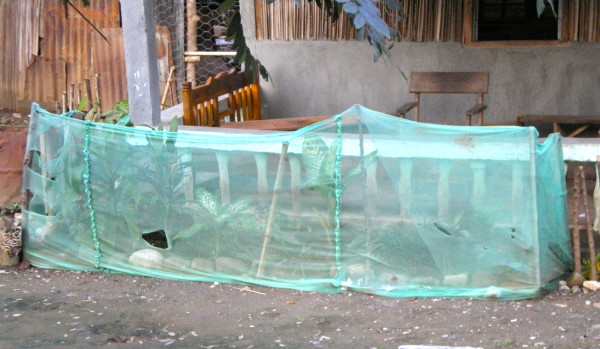
**Alternative use of ITNs for garden protection, Covalima District**.

Nets were also observed that had been fashioned into several styles of fishing nets; that were being used to protect vegetable crops in home gardens, and being used to protect saplings in an orchard (Figures 4 and 5). Alternative usage of nets was directly observed in Dili (fishing nets) and Covalima (fishing nets and crop-protection).

In Manatuto, one new net that the head-of-household reported purchasing for $15 USD at a local market had a clearly visible, but defaced, Ministry of Health hangtag from antenatal clinic distribution programmes. A visit to this same market, and visits to several other markets in different districts, did not produce evidence that this is widespread, but the presence of foreigners may have made vendors hesitant to show any nets they might have had for sale. The nets for sale in all the markets were untreated, conical shaped nets. Informal interviews with shopkeepers suggested that the differences between treated and untreated nets are very poorly understood; indeed, several shopkeepers in Dili were even unaware about the existence of treated nets.

Several participants noted that the treated nets were actually preferable to commercial fishing nets, because the permethrin immobilized small fish and shrimp, making it easier to catch them. Several NGO workers also reported hearing this in other districts, and the practice appears to be fairly widespread in Timor-Leste. The impact of free, antenatal clinic distributed nets on this type of re-purposing is unclear. In Covalima, there was widespread use of nets as protective barriers for garden crops and tree seedlings. Observation in the region confirmed many *new *nets were being re-purposed for gardens and fishing; "filtering the corn" and filtering salt were also mentioned, but the benefits of these practices are unclear. However, several respondents in Manatuto reported that they only used old or worn out nets for other purposes; it was also reported that they were only used for other purposes after being chewed by mice.

Similar alternative net uses have been documented in sub-Saharan Africa, but in a similar study in the Solomon Islands this was only a minor issue reported by a few participants [28,29].

### General malaria knowledge, causes and treatments

Many areas had very good knowledge of malaria, due to extensive education campaigns from the Ministry of Health; several participants even mentioned "*falciparum" *and *"vivax*" specifically, and were knowledgeable about the relative severity.

However, there was wide-ranging confusion about the causes of malaria. Almost all participants knew that mosquitoes caused infections, but a wide range of other factors were also believed to cause malaria; this was less prevalent in Dili. These ranged from simple sanitation issues (trash, flies, faeces, etc.) to a pervasive belief among both men and women that hanging or folding laundry incorrectly also caused malaria. The basis of this belief is unclear, but could be related to the swarming of insects around wet clothing.

When asked to list the symptoms of malaria, the responses were generally non-specific, including fever, headache, chills, "feeling trembling," along with some ambiguous responses, like backaches and stomach aches.

### Risk perception and seasonality of infections

A wide range of opinions was voiced in the focus groups. It was almost universally accepted that malaria was serious during the rainy season in all the studied regions, but many also thought it was year-round. A few participants mentioned the dry season as being the worst, as well; but there were no specific geographic trends in responses.

When asked how often people in their community had malaria (confirmed or not), groups in Dili and Manatuto said it was constant, while one group in Covalima said essentially never. However, this was a village along a fairly busy road, with good access to a primary health centre. In more rural isolated sites in Manatuto and Covalima districts, "everyone" was the most common response.

The single exception to this theme was the final mixed-gender focus group (Covalima district), where it was reported that there had been no malaria in the village for as long as anyone could remember. The facilitator asked a series of follow-up questions, and it was confirmed that this was indeed the case. However, this information was in contrast to reports from the district health staff, who admitted that all the areas in the district had a high prevalence of infections. It is possible that there was some reporting bias in this last group, or confusion about malaria versus other febrile illness.

### Non-users

When asked which family members do not generally sleep under nets, one group was consistently mentioned: young boys who sleep at their friends and neighbour's houses, on porches or verandas. This appeared to be common practice in many areas, occurring on a fairly regular basis (1-2 nights each week). This could lead to significant and regular exposure to vectors in a vulnerable group. Others respondents mentioned a range of household members including husbands, some of their children, and younger siblings, but no simple patterns emerged.

### Other protective measures

Many participants used a range of other tools for mosquito control. Commercial brands of repellent, insecticides, and coils were commonly mentioned, along with building smoky fires from specific types of wood or dung. Use of commercial products was more common in Dili and Manatuto than Covalima districts. Wearing long-sleeved clothing, long pants, and blankets were also mentioned by a few groups.

A single focus group in Covalima also mentioned it was common in the village to chew a very bitter leaf that helped to cure malaria, which the facilitators believed to be *Cinchona *sp., based on the plant name in Tetun. Some references suggest cultivation of various *Cinchona *species for medicinal quinine in Timor since the Portuguese era, but it unclear how common this practice of self-medication might be [30].

### Care-seeking behaviour

Care-seeking behaviour was highly divergent based on the geography of the study groups with distinct differences between rural and urban areas. In Dili, and per-urban Manatuto, almost all participants stated that a blood test at a health clinic was the first step if they suspected they had malaria; one female participant in Manatuto even said that all members of the community should tell others if they should get care, since everyone could be affected.

Several groups also specifically mentioned "the orange pills where you have to take four tablets a day" (Coartem^®^, Novartis, the standard of care in Timor-Leste). These areas also cited information from healthcare workers and TV campaigns, and many participants had quite sophisticated knowledge of blood tests, mentioning the number of "stars" (clinical reporting scale for parasitaemia levels).

In rural Manatuto and Covalima, the respondents said they would use paracetamol or herbal remedies, or use various leaves "chewed to pieces and mixed with hazelnuts, then boiled and affixed to the forehead" for many symptoms, including fever. It was reported that these traditional remedies were used first, and then if the patient did not improve, they would only then seek care at the primary health clinic or hospital. Women in rural Covalima specifically mentioned the prohibitive cost of $1 USD for arranging motorbike transportation to a health clinic. This barrier led to either a long delay on care-seeking, or a two-hour walk to a health clinic for confirmation and treatment.

### Comparison with other studies

The complex range of behaviours reported in Timor-Leste are overall comparable to those found in similar studies in other developing countries [31-33]. Specifically, the confusion about causes of malaria, use of traditional remedies, and delayed care-seeking are common features of most reports. The most noteworthy differences are the confusion about who can sleep under nets (including separation of couples), and the very widespread re-purposing of nets in the study districts. This direct observation of widespread alternative use (while not from a defined sampling frame) provides an evidence base for "mis-use" and is a crucial component of understanding the complex behavioural economics of net usage, in Asia as well as sub-Saharan Africa [34].

The study that is most analogous to the situation in Timor-Leste is a recently published qualitative study from the Solomon Islands [29]. In this study, the primary impediments to usage were flexible sleeping arrangements (young boys and adult men often sleeping at the house of friends and neighbours); a general perceived lack of net effectiveness; and a perception in many areas that malaria risk was very low.

In Timor-Leste, CARE International performed a post-interventional study as part of a monitoring and evaluation exercise. This survey included a single open-ended question about the reasons for not using bed nets, but without detailed probing follow-up queries. The two main barriers in both intervention and non-intervention areas were reported to be insufficient nets within the household, and nets being too hot to sleep under [35].

### Strengths and limitations

This study is one of the first undertaken to explore the attitudes and daily practices of the high-risk populations of the Southeast Asia/Pacific region, and probes behaviours relating to a pressing and very significant public health issue. Secondly, it is one of the few qualitative studies of bed net usage in Asia, and adds to the results from studies in Vietnam, the Solomon Islands, and India [20][29][36].

Qualitative studies by definition are non-representative, and the presence of outsiders/foreigners may have affected responses. A single focus group was run as a mixed group; it was planned as a male group, but most men were needed for rice harvesting. This was a significant problem in several areas, as the participants who could readily attend the groups tended to be older, as the working-age groups were occupied in the fields, leading to a somewhat older cohort than originally planned. However, as childcare is a multi-generational effort in Timor, these participants' opinions are quite important, and reflect relevant information about household decision processes.

Explicit notice was given about not compensating participants for their participation aside from snacks and water, and it was explained that participation would be helping to improve the health in their entire community. However, several participants still asked if there would be any net distribution or health clinic activities. Specific participants dominated several discussions, and lastly, the study organizers had no direct control over recruitment of participants.

One persistent issue in qualitative research is a balance between reporting illustrative responses, and the need to highlight any unusual or surprising opinions [22]. Focus groups by their nature are not representative of the population as a whole, so responses may be very important social themes, or simply one individual's idea or understanding.

## Conclusions

This study explores some of the main barriers and hindrances to optimal bed net usage in Timor-Leste. The results have been shared with both the Ministry of Health and the National Malaria Control Programme, and will allow more targeted and effective behaviour change materials to be developed. Further qualitative and quantitative studies will be necessary to understand several factors: the true extent, behavioural economics, and potential health impacts of alternative net usage; the direct impact of the common misunderstanding about who should use nets; and a more detailed exploration of families' preferences about net design. These factors will be crucial in achieving widespread consistent and correct usage, as Timor-Leste make progress towards universal coverage and eventual malaria elimination.

## Competing interests

The authors declare that they have no competing interests.

## Authors' contributions

AAL and BAS conceived, planned and organized the study. AJA organized and facilitated the fieldwork. AAL analysed the data, and wrote the first draft. AWS helped to shape the analytic framework. AWS and BAS revised the manuscript.

All authors read and approved the final manuscript.
